# The Chosen Aspects of Skew Rolling of Hollow Stepped Shafts

**DOI:** 10.3390/ma14040764

**Published:** 2021-02-06

**Authors:** Jarosław Bartnicki, Yingxiang Xia, Xuedao Shu

**Affiliations:** 1 Faculty of Mechanical Engineering, Lublin University of Technology, 36 Nadbystrzycka Str., 20-618 Lublin, Poland; 2 Faculty of Mechanical Engineering and Mechanics, Ningbo University, Ningbo 315211, China; xiayingxiangnbu@163.com (Y.X.); shuxuedao@nbu.edu.cn (X.S.)

**Keywords:** skew rolling, hollow stepped shafts, process limits

## Abstract

The paper presents chosen aspects of the skew rolling process of hollow stepped products with the use of a skew rolling mill designed and manufactured at the Lublin University of Technology. This machine is characterized by the numerical control of spacing between the working rolls and the sequence of the gripper axial movement, which allows for the individual programming of the obtained shapes of parts such as stepped axles and shafts. The length of these zones and the values of possibly realizable cross-section reduction and obtained outlines are the subject of this research paper. The chosen results regarding the influence of the technological parameters used on the course of the process are shown in the present study. Numerical modelling using the finite element method in Simufact Forming, as well as the results of experimental tests performed in a skew rolling mill, were applied in the conducted research. The work takes into account the influence of cross-section reduction of the hollow parts and the feed rate per rotation on the metal flow mechanisms in the skew rolling process. The presented results concern the obtained dimensional deviations and changes in the wall thickness determining the proper choice of technological parameters for hollow parts formed by the skew rolling method. Knowledge about the cause of the occurrence of these limitations is very important for the development of this technology and the choice of the process parameters.

## 1. Introduction

One of the solutions that make it possible to reduce the weight of the structure while maintaining similar mechanical characteristics is to replace filled products with hollow ones. Hollow elements such as axles and shafts, while maintaining similar dimensions and functional features, also allow a reduction in the consumption of materials necessary for their production and enable a reduction in the amount of waste generated during their processing. This is important for the protection of the natural environment, respect for the use of natural resources and reduction of energy consumption. In the field of plastic working, the formation of this type of product is possible with the use of various manufacturing technologies [1,2]. Unfortunately, in most cases, the profitability threshold is to start with large-scale or mass production, below which all activities in this area are devoid of purpose.

The introduction of hollow elements such as axles and stepped shafts as machine parts, in addition to reducing the weight of the structure, allows for the reduction of the moments of inertia which, for example, determine the performance of selected mechanisms equipped with them. Such activities, however, require the optimization of the shape of this type of product in terms of changes in both diameters and wall thickness along their length [3,4]. Currently, elongated axisymmetric forgings with lengths of up to several hundred millimeters are produced by cross-wedge rolling (CWR) technology [1,2] or forging longitudinal rolling (mainly blanks intended for forging) [3,4,5].

One of the solutions for rolling products in medium and small series is the use of the CWR technology with the use of flat wedge tools. This significantly reduced the cost of the tooling. Attempts to solve the problem of the idle return of tools to the initial position by introducing cross flat wedge rolling in two directions eliminated this limitation [6]. Unfortunately, the cost of building a machine equipped with such a complex kinematic system was in contradiction with the advantages of the proposed solution for smaller production series. On the other hand, long forgings (with lengths up to several meters) are manufactured on the basis of the open-die forging processes (forgings produced individually or in small series) and forging with swaging machines [7,8,9,10] (forgings with increased accuracy). Considering small elongated forgings (with lengths up to 200 mm), very efficient rolling processes can also be applied in skew rolling mills equipped with rolls with screw blanks [11,12]. These methods allow for the production of (most often) semifinished products for further mechanical processing [13]. They are also used as semifinished products for die forging [14] or extrusion [15].

Solutions enabling the practical implementation of the production processes of hollow products, such as stepped axles and shafts, usually require significant costs to start their production. An additional difficulty in this field is the necessity to constantly narrow the manufacturing tolerances associated with limiting the allowances for finishing machining or switching to manufacturing in standards that allow its partial exclusion. Actions taken in this area lead to a significant increase in the participation in the design of these types of processes by research centers dealing with numerical simulations and designing technological processes, which brings measurable results. The results of the research related to it, however, are still not broadly reflected in the processes of forming hollow products intended for small-lot production.

The presented works in the field of forming hollow products with the direct possibility of implementation into small-lot production concern, among others, rotary compression technology [16], where the obtained filled or hollow product is rolled by approaching rotating roller tools. This method, apart from offering significant advantages, has a limitation related to the modeling of the wall thickness of the resulting hollow product. Wall thickness changes according to the solution provided are made by determining the ratio of the rotational speed of the tools to the speed of their approach to each other. The change in the kinematics of the metal flow, which then takes place, allows a certain adjustment of the wall thickness of the obtained product, and, however, concerns its entire length.

Similar limitations apply to the cross-wedge rolling and rolling-extrusion technologies for hollow part forming presented in the earlier works of Lublin University of Technology [17,18]. An important advantage of the new solution is the possibility of forming various shapes of products without changing tools, using only the change of the speed of the movement of the tools in relation to each other. The results of the research work carried out earlier on this forming process are presented in [19], where design assumptions are given and the selection of technological parameters enabling the practical implementation of the process for selected shapes of products is presented. The favorable kinematics of the material flow, which allows for a significant reduction of the cross-sections of the elements with a limited risk of crack formation, is a very important feature of the presented solution. The proposed technology of skew rolling allows us to bypass this limitation, which is one of the goals of the conducted research.

The presented stage of the research carried out included the numerical analysis and the experimental verification of the skew rolling process of hollow parts with different technological parameters. The aim of the tests was to determine the possibility of rolling hollow forgings, the kinematics of the material flow and the limiting phenomena that may occur in this process. Knowledge of these data allows us to plan further experiments in the extended range of technological parameters. As a consequence, it allowed us to determine their influence on the dimensional accuracy and changes in the wall thickness of the resulting products. The information collected in the course of ongoing research provides guidelines for determining the limitation of the efficiency of the skew rolling process of hollow products due to the loss of stability and the practical value of technological allowances.

## 2. Skew Rolling Technology

**Figure 1:**
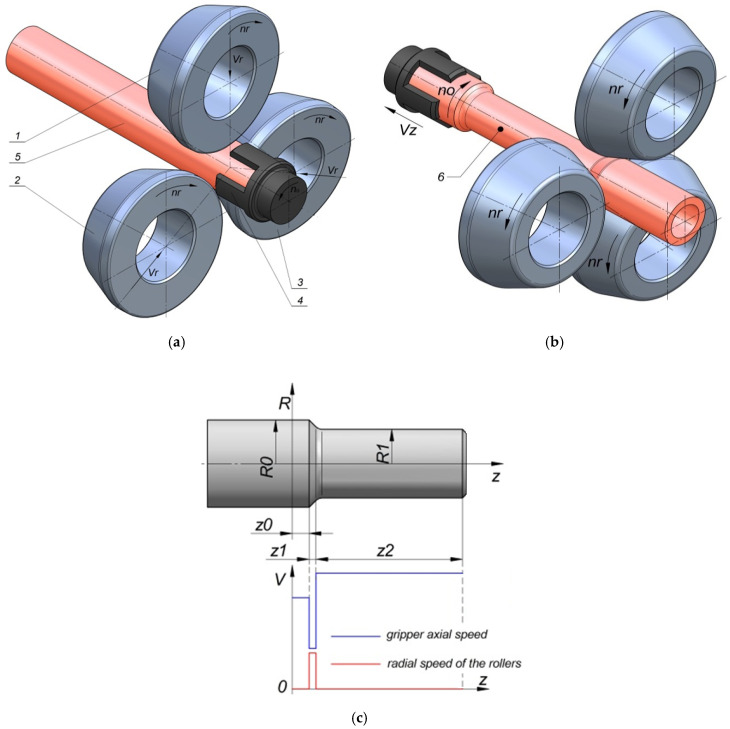
Scheme of the skew rolling process of stepped shafts in a three-roll skew rolling mill with the controlled movement of the tools and the billet: (**a**) the beginning of the process, (**b**) the advanced phase of the process, (**c**) changes in the radial velocity of the tools and the axial velocity of the billet based on the shape of the rolled product; 1, 2, 3—rolls, 4—gripper, 5—billet, 6—formed part.

The technology of skew rolling consists of introducing the billet into the workspace between three rotating rollers, the rotation of which sets the rolled product in motion. In addition to changing the spacing of the rollers during the process, the gripper changes the longitudinal position of the billet in relation to them. The movement of this tool is correlated with the sequence of radial movements of the rollers, which makes it possible to form steps with selected cross-section reductions and lengths (Figure 1).

The modernization of the aggregate and further works related to the rolling of filled and hollow products aimed at improving this solution are successively carried out. Due to the possibility of presenting an implementation offer for the skew rolling of stepped hollow products, it was decided in the current work to check selected variants of forming in terms of wall thickness distributions and obtained dimensional deviations.

## 3. Materials and Methods

**Figure 2:**
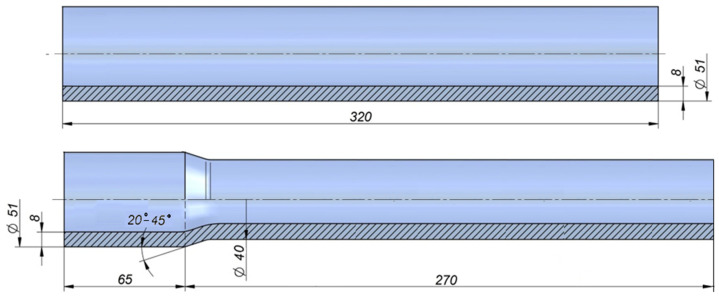
Hollow billet and the example of finished part (dimensions in mm).

**Figure 3:**
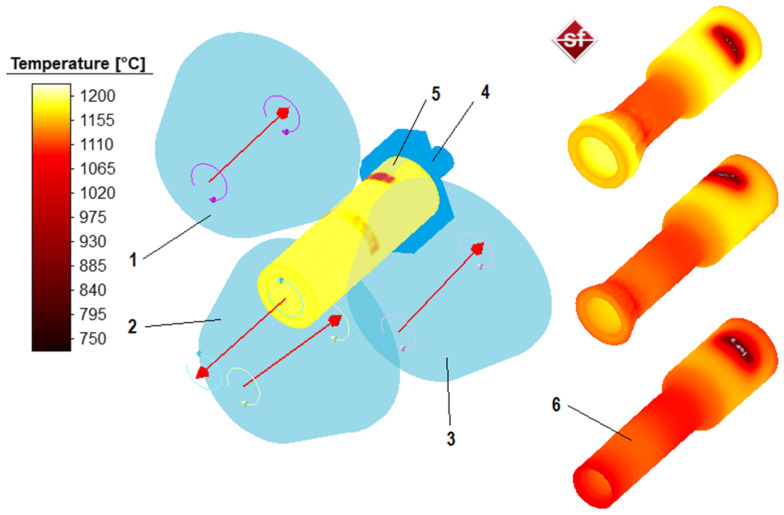
The geometrical model of the skew rolling process of the stepped shaft forming developed for the FEM analysis: 1, 2, 3—rolls, 4—gripper, 5—billet; 6—formed part and distribution of temperatures (step reduction from Ø51 to Ø30 mm, gripper velocity 20 mm/s).

The finite element method in the Simufact Forming 15 software (v.15.0.0.60847) was used for the simulations. During simulations the rolling process of hollow shaft forgings was analyzed, the shape and dimensions of which are shown in Figure 2. For the purposes of the analysis, geometric models of the skew rolling process of hollow shafts were developed (Figure 3). Each model consisted of three identical tools—conical rolls (1, 2, 3), a gripper (4) and billet (5).

For rolling hollow forgings, the billet was a pipe section with a diameter of Ø51 mm, wall thickness t = 8 mm and length 220 mm. It was assumed in calculations that the billets are made of S355 steel, which were modeled as rigid plastic objects using eight-node cubic elements of the first order. The material model of S355 steel was taken directly from the Simufact Forming software library and was described by the Equation (1) [20,21]:

(1) $$\sigma_{p}=2549.49\cdot e^{\left(-0.0033907\cdot T\right)}\cdot \varepsilon^{\left(-0.00032304\cdot T+0.19313\right)}\cdot e^{\left(\frac{-0.000049038\cdot T+0.011774}{\varepsilon }\right)}\cdot {\dot{\varepsilon }}^{\left(0.00011724\cdot T+0.043833\right)}$$

where: *T*—temperature (in the range from 700 °C to 1250 °C), *ε*—strain, $\dot{\varepsilon }$—strain rate.

Similar relationships of the stress, strain and strain state for the given steel were obtained in the tests [22], where the research methodology was fully presented.

For further industrial research for the selected hollow product, a material model developed independently on the basis of plastometric tests will be used. The given material is widely used in welded steel structures and machine components, for example in the agricultural sector. The results obtained with the use of a given steel model indicate the parameters that can be used for a wider range of used steels, including those with a slightly higher carbon content. The use of the given material was also caused by its wide commercial availability.

It was also assumed that for all the analyzed cases at the initial stage of the process the entire billet material was heated to 1200 °C, whereas the tools retained constant temperature 100 °C throughout the entire process. During the process the rolls rotated in the same direction at the constant velocity *n* = 60 rotations/min and the gripper, in which the billet was mounted, was free to rotate about the rolling axis. The velocity table (Table 1) was used to define the movement of the tools in in the radial direction and the gripper in the axial direction so as to obtain the assumed outline of the formed parts. Additionally, in the process, the value of the tool inclination angle was set to 5.0° and it was combined with two values of reduction of the diameter of the formed initial step Ø51 mm to Ø30 and Ø40 mm. The model of constant friction was used to describe the contact surface of the formed material and tools. Due to the fact that the skew rolling process was conducted in hot working conditions, the friction factor assumed in the calculations was relatively high, *m* = 0.8 [21]. Moreover, it was assumed that the heat transfer coefficient for the couple material-tools was equal 20 kW/m^2^ K, whereas for material-environment 0.35 kW/m^2^ K.

**Figure 4:**
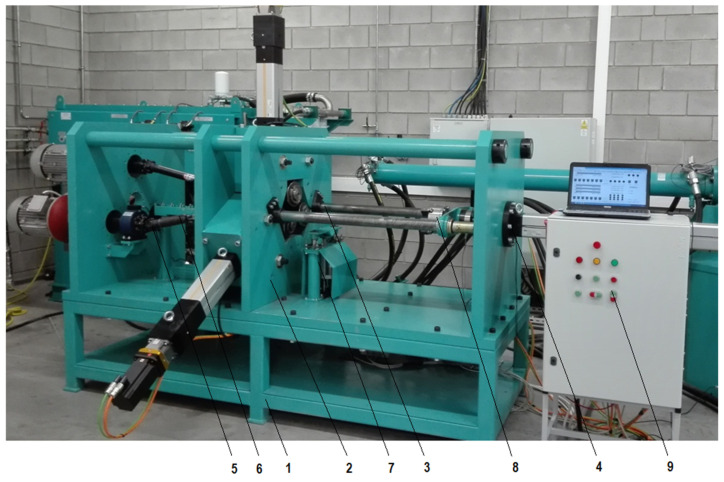
The skew rolling mill with numerically controlled movement of rolls and gripper: support frame—1, drive unit—2, work roll cage—3, axial shift unit—4, drive transmission unit—5, batch support unit—6, the gripper group—7, the rolled forging support unit—8, and the power and control system—9.

The research was based on numerical modelling using finite elements method (FEM) as well as experimental testing, performed in laboratory conditions in a numerically controlled skew rolling mill shown in Figure 4. During rolling, the billet is placed in the head of the support unit. Then, its one end is secured in the four-jaw holder of the gripper assembly. After that, the axial feed electro-screw actuator moves the billet together with the gripper assembly to the initial position. Then, the rotary movement of three tool shafts is started with tool rolls mounted on their mountings. In the next step, the three sliders start to move in radial direction, which is caused by electro-screw actuators. At the same time, the movement of the electro-screw axial slide actuator is initiated, which causes axial movement of the gripper assembly together with the rolled billet towards the axial shift actuator assembly as a result of the action of the tool rolls rotating in the same direction on the workpiece is its rotation. At the same time, the radially moving tool rolls reduce the cross-section of the formed steps. Numerically controlled skew rolling mill is characterized by a block structure and consists of nine basic units (Figure 4).

The proposed solution for rolling products with diameters up to about 80 mm is implemented using tools with a diameter of 150 mm and a radius of rounding the edge of the rollers R3. For the given diameter of the rollers, a certain compromise was obtained between the values of unit pressures on the formed material, torques necessary to ensure the machine drive system and the efficiency of the process. Further research in this area will be continued in a wider range of diameters of rolled billets and the corresponding diameters of working tools. It should be mentioned here, however, that increasing the diameter of the rollers leads to a rapid increase in the value of the torques, which forces significant structural changes in the construction of the aggregate and increases the costs of its production and operation. With regard to the radius of rounding of tools in the process of forming stepped products, the proposed values are a compromise between the attempt to obtain steeper steps and the formation of characteristic stairs in the transition zone. Increasing the value of the radius of the rounding of the rollers allows the use of higher forming speeds with simultaneously extended transition zones of the products. Reducing the value of the fillet radii forces the use of lower feed rates per revolution, which leads to a reduction in the efficiency of the process and excessive cooling of the product in the entire volume. Moreover, it limits the geometrically minimum diameter of the rolled forgings that can be obtained by this method.

## 4. Results

**Figure 5:**
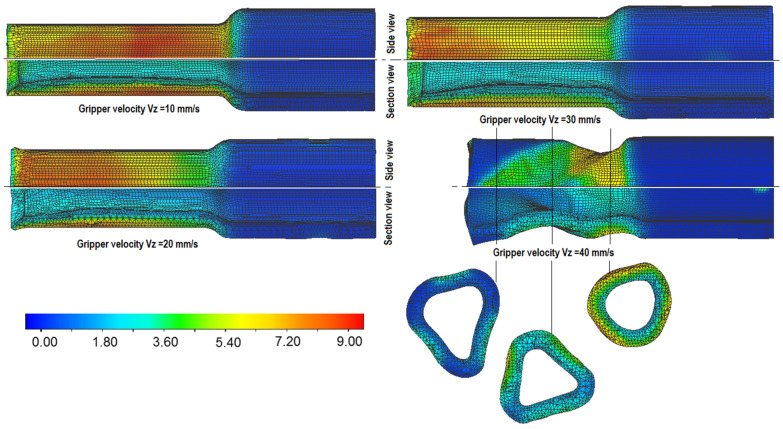
The progression of shapes and effective strain distributions in hollow shafts skew rolled with different gripper velocity.

The described coefficients related to the heat exchange were included in the calculations. An example of product temperature changes during rolling is given in Figure 3. The obtained values were comparable to those obtained during measurements in laboratory tests. Figure 5 shows the results of the numerical calculations for the hollow parts formed at the given values of the velocity of the gripper’s movement. In this figure the progression of the product shape in the side view and in the longitudinal section are also compared. For the case with the highest velocity of the gripper’s movement, the occurrence of process stability disturbances in the form of triangulation related to the material sliding in relation to the rolls was observed. This phenomenon is equivalent to the ovalization occurring during the rolling of products with two tools at too high values of reduction ratios or wedge forming angles in the case of cross-wedge rolling [3].

Additionally, considering the changes in wall thickness in the longitudinal section of the analyzed products, it was noticed that with the increase in the forming velocity, the thickness distributions of this parameter fluctuate more. It is particularly easily visible for the example given in Figure 5 for the gripper’s velocity of 30 and 40 mm/s. Taking into account the values of the intensity of effective strain, it can be concluded that at lower velocities of the gripper’s movement, the value of the strain increases. It is connected with the increasing share of redundant strain in the process. An increase in the length of the formed steps, caused by the material flowing in axial direction mostly in surface layers, can also be observed. Because of that the front surfaces of the formed steps become concave. In many cases, however, even a slight change of the parameters causes the products to be deformed. The billet wall thickness, the reduction of diameters and the relative tools’ velocities are among the basic technological parameters acting on the skew rolling process and the quality of the finished parts.

**Figure 6:**
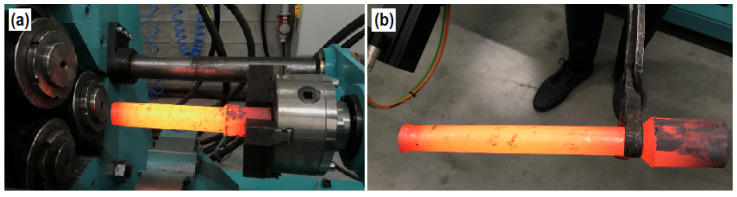
Obtained in experimental verification hollow parts: (**a**) with diameter reduced to Ø40 mm placed in gripper and showing skew rolling mill workspace; (**b**) part with diameter reduced to Ø30 mm.

Tests of the rolling process of hollow products with diameters analogous to those in the numerical tests were carried out by means of the presented machine, with the use of billets 100 mm longer (320 mm in place of 220 mm in FEM analysis). The products were rolled with the gripper’s velocities of 20, 30 and 40 mm/s, achieving reduced step diameters of 30 and 40 mm (Figure 6). The analysis of the results obtained experimentally was based on the results achieved with manual measuring tools. Due to that, the measurement accuracy was assumed at the level of 0.05 mm, and the measurements were made many times, excluding intermediate values. The values obtained in the numerical analysis were measured between the extreme nodes of the FEM mesh in the selected area. In both cases (diameter deviations and wall thickness changes), subsequent measurements were made in cross sections spaced every 5 mm of the length of the rolled product.

**Figure 7:**
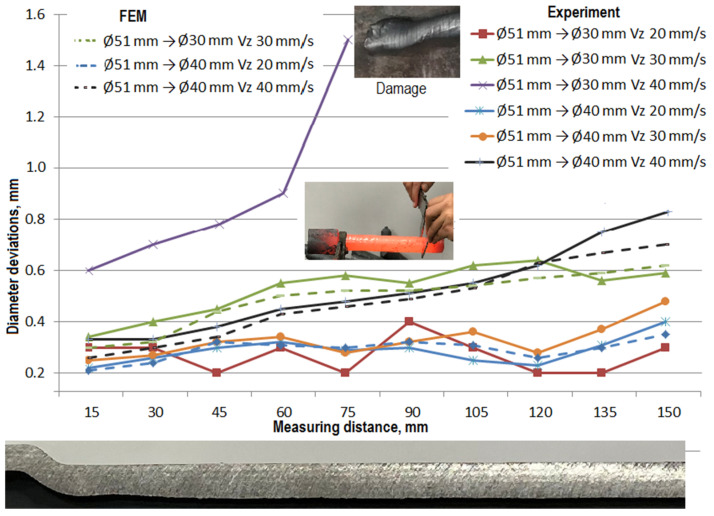
Changes of hollow part reduced diameter deviations and wall thickness distributions depending on presented skew rolling process parameters.

**Figure 8:**
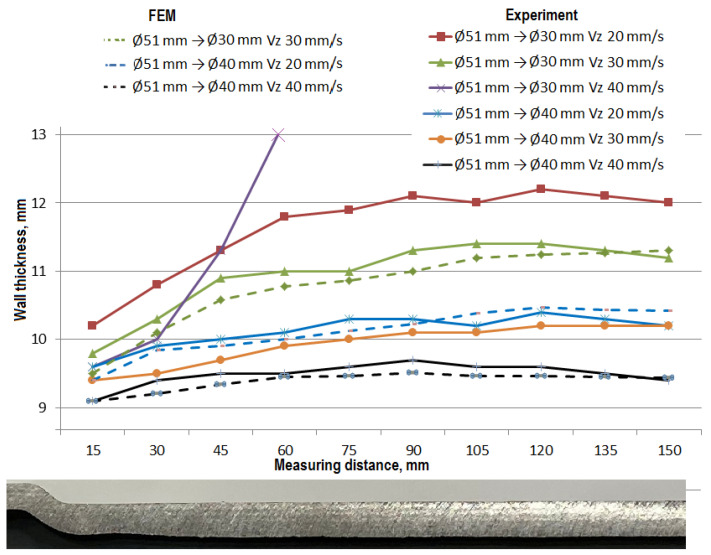
Changes of hollow part wall thickness distributions depending on presented skew rolling process parameters.

The results of the tests carried out are summarized in the figures illustrating the dimensional deviations of the diameter (Figure 7) and the courses of changes of the experimentally obtained part wall thicknesses (Figure 8).

In order to minimize the dimensional tolerances in this process, the obtained results were compared with numerical results and used in determining the recommendations for the implementation of the rolling process of hollow parts with steps. On the basis of the obtained results, it was found that the smallest dimensional deviations in the rolling process of hollow parts with the reduction of the step diameter by approximately 40% (Ø51 mm reduced to Ø30 mm) resulted in the use of the gripper movement velocity of 20 mm/s, and with a reduction of approximately 20% (Ø51 mm reduced is Ø40 mm) this velocity can be increased to 30 mm/s.

## 5. Discussion

The results of the forming of hollow products obtained as a result of numerical simulations have been verified in experimental tests. A direct comparison of the obtained results concerning the appearance of the loss of shape stability in the form of triangulation was confirmed in both analyzed cases with an increase in the linear speed of the billet and a greater reduction of its cross-section.

Correlating the given values with the velocity of rotation of the rolls *n* = 60 rotations/min results in obtaining the value of the feed rates at the level of 2 and 3 rotations per 1 millimeter of gripper displacement, respectively. In the case of formed products with the gripper movement velocities of 40 mm/s (1.5 rotations per 1 millimeter), both in numerical calculations (Figure 5) and in experimental tests (Figure 7 and Figure 8), the formed product with Ø30 mm lost wall stability and was destroyed. For the proposed value of the diameter reduction, there were significant diameter deviations (over 0.8 mm). The changes in diameter allowances (Figure 7), related to the applied process parameters shown in the figure indicated the presence of strong relationships between the feed rates, the length of the step being formed and the cross-section reduction ratios. Thus, along with the distance from the beginning of the formed step, the shape deviation values gradually increase, which is related to the increase in the free length of the product.

Similar observations apply to increasing the feed rate, where it also directly influences the increase in this parameter value. The changes in the values of deviations measured on the length of the reduced step related to the technological allowances that it is necessary to add are so small that in the scope of the standards for the acceptance of hot-formed products, they can be omitted. Their causes, however, can be seen in the inhomogeneous flow of the material during the process or tolerances in the commercial batch material. The surface quality of the product in the conical zone corresponds to the accepted hot forming conditions. The assumed technological allowances related to the emerging steps are smaller than 0.3–0.4 mm. However, their appearance may also be the result of the use of weaker electric actuators in the aggregate, which operated close to the maximum range of the force parameters. The current modernization involving the reconstruction of the control system and the change of the roller track with the use of hydraulic cylinders with much higher values of generated forces should improve this situation.

The changes in the wall thickness values presented in Figure 8 indicate their stable course along the length of the shaped step of the hollow product. However, the feed rate value is of particular importance in this case, as the obtained increases in wall thickness depend on it. With lower feed rates, the wall thickness increases, and vice versa, until the product stability is lost. This phenomenon has already been analyzed in previous studies on rotary compression and rolling extrusion processes [2,5,16], where the changing proportions of the axial and radial flow of material in these processes were analyzed. In the process of skew rolling of hollow products, this phenomenon is analogous, but the wall thinning does not occur in any of the examined cases. Reducing the feed rate speed in order to increase the wall thickness unfortunately results in a significant increase in the duration of the process.

## 6. Conclusions

The possibility of the skew rolling of hollow parts with the use of numerically controlled skew rolling mill is presented. The numerically determined and experimentally verified technological parameters of the step forming of hollow parts indicated the practical scope of using this method in the production of exemplary stepped hollow shafts. It has been shown that with the increase in the velocity of movement of the gripper in relation to the rotation of the rolls, the dimensional deviations on the diameter of the step created increase. Similarly, the elongation of the rolled product increases, yet a relatively smaller increase in the wall thickness is observed. When the forming velocity is reduced, the material in the formed area begins to flow towards the workpiece axis, which results in an up to 1.5-fold increase of wall thickness. Within this range of parameters, the lowest dimensional deviations on the diameter of the reduced step are also observed, which may significantly reduce the necessity of machining in the finishing treatment of hollow shafts manufactured in this way. However, after exceeding a certain limit, a sharp increase in shape deviations occurs, leading to the destruction of the product. The approximate ranges of technological and geometric parameters given in the test results provide guidelines for the design of processes for selected stepped hollow shafts.

Further research in the given range will be carried out in relation to the rolls’ skew angles and the temperature ranges to which the billet is heated, which will also be important for the complex metal flow mechanisms in the tested process. High efficiency, simple construction, the possibility of forming products from tube-shaped billet without the replacement of tools and easy automation constitute the major advantages of the proposed technology. It is possible to manufacture the products both in small series and in mass production thanks to the simple construction of the machine and tools. Furthermore, in the case of skew rolling in hot conditions, problems with removing scales do not occur, since they fall down under gravity between the rolls. Due to the facts presented above, the described technology using numerically controlled tool movements is very attractive for the industry.

## Figures and Tables

**Table 1:** The velocity and geometrical parameters selection for numerical analysis (directions R and Z according to Figure 1).

Inclination Angle			5°	
Reduced Diameter			30 mm	40 mm
Movement	Roll 1; 2; 3	R direction	(0 s–5.25 s) 2 mm/s	(0 s–2.75 s) 2 mm/s
			(5.251 s–15 s) 0 mm/s	(2.751 s–13 s) 0 mm/s
	Gripper	Z direction	(0 s–2.75 s) 2 mm/s	(0 s–2.75 s) 2 mm/s
			(2.751 s–end of process) 10; 20; 30; 40 mm/s	

## Data Availability

Not applicable.
